# A Traditional Chinese Medicine Formula Danshen Baibixiao Ameliorates Imiquimod-Induced Psoriasis-Like Inflammation in Mice

**DOI:** 10.3389/fphar.2021.749626

**Published:** 2021-12-03

**Authors:** Xiaoqi Jin, Hongfeng Xu, Chuanqi Huang, Haoran Ma, Xin Xiong, Lu Cheng, Fuqian Wang, Yan Feng, Geng Zhang

**Affiliations:** ^1^ School of Pharmacy, Hubei University of Chinese Medicine, Wuhan, China; ^2^ Department of Pharmacy, Wuhan Hospital of Traditional Chinese and Western Medicine, Wuhan, China; ^3^ Department of Pathology, Wuhan Hospital of Traditional Chinese and Western Medicine, Wuhan, China

**Keywords:** traditional Chinese medicine, IL-23/TH-17 axis, NF-κB, STAT3, MAPK

## Abstract

**Background:** Danshen Baibixiao (DB) is a traditional Chinese medicine formula, which has been used to treat psoriasis for decades. Although DB shows good efficacy in clinical practice, the pharmacological effects and underlying mechanisms of DB remain elusive. This study aimed to evaluate the anti-psoriatic effects of DB and explore its underlying mechanisms in an imiquimod (IMQ)-induced psoriasis-like mouse model.

**Materials and methods:** DB was orally administered on IMQ-induced psoriatic mice. Psoriasis area severity index (PASI) was used to evaluate the severity of the inflammation in skin, and histological changes were evaluated by hematoxylin and eosin (H and E) staining. Levels of inflammatory cytokines, such as tumor necrosis factor *α* (TNF-α), interleukin (IL)-17A, IL-23, IL-6, IL-1*β* and IL-22 in serum were assessed by enzyme-linked immunosorbent assay (ELISA). mRNA expressions of IL-17A, IL-23, IL-6 and IL-22 were determined by real-time polymerase chain reaction (PCR). Expression levels of proteins related to NF-κB, STAT3 and MAPKs signaling pathways were measured by western blotting (WB).

**Results:** DB significantly ameliorated the psoriatic symptoms in IMQ-induced mice. The serum levels of inflammatory cytokines (TNF-α, IL-17A, IL-23, IL-6, IL-1β and IL-22) were decreased, and mRNA expressions of IL-17A, IL-23, IL-6 and IL-22 in skin tissues were down-regulated. Moreover, WB analysis indicated that DB inhibited the activation of NF-κB, STAT3 and MAPKs signaling pathways.

**Conclusion:** This study confirms the anti-psoriatic activity of DB in IMQ-induced psoriasis-like mice. The possible mechanism may relate to the activities of regulating the IL-23/TH-17 axis and suppressing the activation of NF-κB, STAT3 and MAPKs signaling pathways.

## Introduction

Psoriasis is a chronic auto-immune inflammatory disease, usually manifesting silvery scales, thickened *epidermis* and erythema on different parts of body. Pathologically, it is characterized by abnormal proliferation and differentiation of keratinocytes, infiltration of inflammatory cells and release of inflammatory cytokines (Greb et al., 2016; Chiang et al., 2020). It is reported that psoriasis affects approximately 2,3% of the global population (Armstrong and Read, 2020). What’s worse, patients with psoriasis often suffer from comorbidities, such as cardiovascular disease, metabolic syndrome, cancer and depression, which synergistically increase the physical and psychological burden on patients (Boehncke and Schön, 2015; Kuai et al., 2020).

Although the pathogenesis of psoriasis is not totally understood, interleukin-23 (IL-23)/T helper 17 (TH-17) axis is believed to be critically involved in the development and maintenance of psoriasis (Gaffen et al., 2014; Schinocca et al., 2021). Upon aberrant activation, dendritic cells (DCs) secrete pro-inflammatory mediators, such as IL-23, IL-6 and TNF-α. TH-17 cells induced by IL-23 and IL-1β, can produce inflammatory cytokines, such as IL-17A, IL-22, TNF-α, IL-6 (Greb et al., 2016; Hou et al., 2018; Rai et al., 2020). These inflammatory factors jointly promote keratinocyte proliferation and other hallmarks of psoriasis. Numerous studies have demonstrated that inflammation related pathways, such as nuclear factor-kappa B (NF-κB) pathways, signal transducer and activator of transcription 3 (STAT3) pathways and mitogen-activated protein kinase (MAPKs) pathways were involved in psoriasis (Haase et al., 2001; Sun et al., 2017; Nguyen et al., 2018; Wang et al., 2019; Xu et al., 2019). NF-κB can mediate gene encoding of many pro-inflammatory and inflammatory cytokines (García-Pérez et al., 2014; Xu et al., 2018). STAT3 is a key transcription factors, participating in T cells’ activation and proliferation, differentiation of keratinocytes and synthesis of inflammatory cytokines (Palanivel et al., 2014). MAPKs, including p38, JNK and ERK1/2, are activated to produce many important inflammatory cytokines, such as TNF-α, IL-23 and IL-6 (Mavropoulos et al., 2013; Hammouda et al., 2020).

Currently, treatment of psoriasis involves topical medicines (corticosteroids, vitamin D analogs and acitretin), systemic drugs (methotrexate, dexamethasone and cyclosporine), phototherapy (UVA and UVB), and biological medicines (TNF-α antagonists, Janus kinase inhibitors, anti-IL-23 agents and anti-IL-17 agents) (Greb et al., 2016). However, none of them can cure psoriasis, and a number of side effects associated with these medications remain a concern (Rapalli et al., 2018; Li et al., 2019b). Therefore, new anti-psoriatic medicine with improved efficacy and safety should be developed. Traditional Chinese medicine (TCM) with a long history of treating psoriasis can be a possible source of new anti-psoriatic medicine.

Danshen Baibixiao (DB), a traditional Chinese medicinal formula comprised of 15 kinds of traditional Chinese medicine ingredients (Table 1), has been used to treat psoriasis for over 30 years in Wuhan Hospital of Traditional Chinese and Western Medicine. The efficacy of DB was widely recognized by doctors and patients. (Xuan and Zhang, 2003; Lin et al., 2005). DB, previously named as Xiaobiwan, was developed by Professor Changfa Hu, a famous TCM doctor specialized in skin diseases. He defined the ingredients and amounts of ingredients used in the formula based on TCM theory and anti-psoriatic medicines recorded in ancient Chinese medicine literatures (Lin et al., 2005). This formula has been developed into a hospital preparation, renamed as Danshen Baibixiao, and has been used to treat psoriasis in the dermatology department of our hospital since 1990s. Among the ingredients, Salvia miltiorrhizae served as the monarch drug of the formula, which was thought to play a major role in the therapeutic action of the formula (Lin et al., 2005). According to TCM theory, Salvia miltiorrhizae has the efficacy to clear blood heat, promote blood circulation and remove blood stasis. Thus, Salvia miltiorrhizae is frequently used in the prescriptions to treat psoriasis (Zhang et al., 2014; Jia et al., 2020). A wide spectrum of secondary metabolites has been identified from Salvia miltiorrhizae. Diterpenoid quinones and hydrophilic phenolic acids are the major bioactive constituents (Su et al., 2015). Several chemical constituents of Salvia miltiorrhizae, such as tanshinone II, danshensu, salvianolic acid B and cryptotanshinone were reported to show anti-psoriatic effects (Li et al., 2012; Tang et al., 2018; Jia et al., 2020; Wang et al., 2020). In clinical observations of DB treatment, total effective rate of therapy with DB was 73.7% (Lin et al., 2005), and total effective rate of combination therapy of DB and phototherapy (UVA) on psoriasis vulgaris was 95.2% (Xuan and Zhang, 2003). Although DB shows good efficacy in clinical practice, the pharmacological effects and underlying mechanisms of DB remain elusive. In this investigation, we evaluated the anti-psoriatic effect in IMQ-induced psoriasis-like mice model and explored the mechanism of DB through its regulation on IL-23/TH-17 axis, and NF-κB, STAT3, MAPK signaling pathways.

**TABLE 1:** Composition of danshen baibixiao.

Ingredients	Source	Medicinal parts	Pocessing method	Mass ratio
Salvia miltiorrhizae radix et rhizome (丹參)	*Salvia miltiorrhiza* Bge	Root and Rhizome	Thick sliced and dried	4
Scorpio (全蝎)	*Buthus martensii* Karsch	Whole body	Dried	2
Zaocys (烏梢蛇)	*Zaocys dhumnades* (Cantor)	Whole body	Segmented and dried	2
Rhei radix et rhizome (大黃)	*Rheum officinale* Baill	Root and Rhizome	Thick sliced and dried	2
Cicadae periostracum (蟬蛻)	*Cryptotympana pustulata* Fabricius	cot	Dried	2
Eupolyphaga steleophaga (土鱉蟲)	*Eupolyphaga sinensis* Walker	Whole body	Dried	2
Cynanchi paniculati radix et rhizome (徐長卿)	*Cynanchum paniculatum* (Bge.)Kitag	Root and Rhizome	Segmented and dried	2
Crataegi fructus (山楂)	*Crataegus pinnatifida* Bge. Var. major N. E. Br	Mature fruit	Thick sliced and dried	6
Isatidis radix (板藍根)	*Isatis indigotica* Fort	Root	Thick sliced and dried	6
Carthami flos (紅花)	*Carthamus tinctorius* L	Flower	Dried	2
Saposhnikoviae radix (防風)	*Saposhnikovia divaricate* (Turcz.)Schischk	Root	Thick sliced and dried	4
Sophorae flavescentis radix (苦參)	*Sophora flavescens* Ait	Root	Thick sliced and dried	2
Phellodendri amurensis cortex (關黃柏)	*Phellodendron amurense* Rupr	Bark	Shredded and dried	4
Scolopendra (蜈蚣)	*Scolopendra subspinipes mutilans* L.Koch	Whole body	Dried	1
Glycyrrhizae radix et rhizome (甘草)	*Glycyrrhiza uralensis* Fisch	Root and Rhizome	Thick sliced and dried	1

## Materials and Methods

### Chemicals and Reagents

Imiquimod cream (containing 5% imiquimod) was obtained from Meheco Keyi Pharma Co. Ltd (Hubei, China, batch number: 190301). Depilatory cream was purchased from Reckitt Benckiser (London, United kingdom). Methotrexate and chemical references (salvianolic acid B, tanshinone IIA, berberine hydrochloride, prim-O-glucosylcimifugin, sophocarpidine, and ammonium glycyrrhetate) were purchased from National Institutes for Food and Drug Control of China (Beijing, China). ELISA kits for IL-17A, IL-23, IL-22, IL-6, TNF-α, IL-1β were purchased from Invitrogen (Carlsbad, CA, United States). Primary antibodies used in western blotting analysis were obtained from Cell Signaling Technology (Danvers, MA, United States).

### Plant Materials and Preparation of DB Extract

All DB ingredients were commercially available TCM decoction pieces, bought from Tianji pharmaceutical company (Hubei, China). The ingredients were authenticated by Professor Hezhen Wu from Hubei University of Chinese Medicine through morphological identification according to Chinese Pharmacopoeia. The specimens of the 15 ingredients were deposited in the herbarium of Wuhan Hospital of Traditional Chinese and Western Medicine for future reference (voucher number from 20190301 to 20190315 for all ingredients). Detailed information on medicinal parts and processing methods of the materials was listed in Table 1. According to traditional use of TCM prescriptions, the ingredients were mixed together and soaked with 10x weight of distilled water, followed by boiled for 1 h. The decoction was filtrated with gauze, and the medicines were boiled with another 10x weight of distilled water for 1 h. The decoctions from the two cycles were mixed and then concentrated to a solution containing 0.228 g/ml of crude drugs with a rotary evaporator (IKA, Staufen, Germany). Concentrated solution containing 0.228 g/ml of crude drugs was used to treat mice of the high-dosage DB group. The solutions of low-dosage (0.114 g/ml) and middle-dosage (0.057 g/ml) DB were diluted from the high-dose solution with distilled water. The solutions were stored at −20°C before use.

The dosages of DB used in our experiment were calculated from human dosage based on body surface area as described by Xu et al. (2002). Body surface area ratio of mouse (20 g) to human (70 kg) is 0.0026. Recommend dose of DB for an adult was 22 g per day. For a 20 g mouse, the equivalent dose of DB was 22 g × 0.0026 ÷ 0.02 kg = 2.86 g/kg, which was set to be the middle dosage in our experiment. The volume of drug administrated to mice was 0.5 ml, thus the concentration of solution for middle dosage was 2.86 g/kg × 0.02 kg ÷ 0.5 ml = 0.114 g/ml of crude drugs. The low dosage and high dosage were set at half or 2 times of middle dosage. Thus, the concentration of the solution for low, middle and high dosage was 0.057, 0.114, 0.228 g/ml of crude drugs, respectively.

### HPLC-MS/MS Analysis

HPLC-MS/MS method was used to analyze the chemical constituents of DB. According to Chinese Pharmacopoeia, salvianolic acid B and tanshinone IIA are used as reference compounds for quality control of Salvia miltiorrhizae radix et rhizome. Berberine hydrochloride, prim-O-glucosylcimifugin, sophocarpidine and ammonium glycyrrhetate are used as reference compounds for Phellodendri amurensis cortex, Saposhnikoviae radix, Sophorae flavescentis radix, Glycyrrhizae radix et rhizoma, respectively (Chinese-Pharmacopoeia-Commission, 2020). And these compounds were reported to be active compounds of these ingredients, respectively (Su et al., 2015; Kreiner et al., 2017; Pastorino and Cornara, 2018; Sun and Lenon, 2019; Zhu and Hou, 2021). Therefore, salvianolic acid B, tanshinone IIA, berberine hydrochloride, prim-O-glucosylcimifugin, sophocarpidine, and ammonium glycyrrhetate were used as standard references. An appropriate amount of each standard compounds were dissolved in a known volume of methanol individually to make the standard stock solutions. The standard working solutions were prepared by diluting the stock solutions with methanol. Then, an appropriate amount of each standard working solution were mixed and diluted with a known volume of methanol to make the mixed standard working solution.

The DB extract was prepared as described above and was filtered through a 0.45 μm filter for the HPLC-MS/MS analysis. The analysis was performed on a Shimadzu LC-20AD liquid chromatography system coupled with an API 4000 mass spectrometer (AB SCIEX, Foster City, United States). A Sunfire C18 column (2.1*150 mm, 3.5 µm particle size, Waters, Milford, United States) was used for the separation of the components. Water and acetonitrile [both containing 0.1% (v/v) formic acid] were used as mobile phase A and B. HPLC analysis was carried out at a flow rate of 0.2 ml/min with a gradient elution (10–30% B from 0 to 20 min; 30–100% B from 20 to 35 min; 100% B from 35 to 40 min).

The quantifications of the chemical constituents of DB were performed with the multiple reaction monitoring (MRM) mode. The turbo spray ion source was operated in the positive ion mode. The source parameters (ion-spray voltage, temperature, gas1, gas2, curtain gas, collision gas) used in the analysis were 5000 V, 500°C, 55, 55, 35, 6 psi. Ion pairs used for multiple reaction monitoring were 741.3/561.3 (salvianolic acid B), 295.3/277.2 (tanshinone IIA), 336.3/321.2 (berberine hydrochloride), 469.1/307.4 (prim-O-glucosylcimifugin), 249.6/148.3 (sophocarpidine) and 840.6/823.6 (ammonium glycyrrhetate). Compounds in the DB were quantified using the standard calibration curves.

### Animals

Male C57BL/6 mice of 20–25 g weight and 8-weeks age were obtained from Laboratory Animal Center of Huazhong University of Science and Technology (Hubei, China). A total number of 60 mice were used in this experiment. The mice were housed in plastic cages under specific pathogen-free conditions of 24–26°C temperature, 55% relative humidity on 12 h light/dark cycles with abundant food and water. All of them were acclimatized for 1 week and kept in good health condition before use. Animal handling and institutional approval were supported and approved by the Institutional Animal Care and Use Committee at Tongji Medical College of Huazhong University of Science and Technology (approval number: 2,520). All experiments were conducted in accordance with the National Institutes of Health Guide for the Care and Use of Laboratory Animals (the seventh edition, United States).

### Animals Treatment and Sample Collection

**FIGURE 1:**
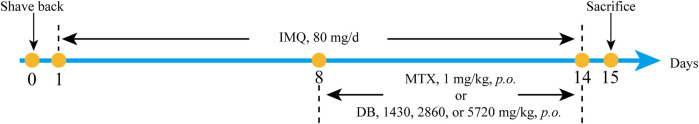
Scheme of the animal experimental procedure.

Backs of the mice were shaved and depilated with depilatory cream to expose the back skin. Sixty mice were randomly divided into six groups (*n* = 10): the normal control (NC) group, the model group (IMQ group), the methotrexate (MTX) treated group, and three groups treated with low, middle and high dosage of DB. Five groups except for NC group received application of 80 mg IMQ cream daily on their back skins for 14 consecutive days. For the last 7 days, the low-dosage DB group (DB-L), middle-dosage DB group (DB-M) and high-dosage DB group (DB-H) received orally 1.43, 2.86, 5.72 g/kg DB (calculated from the human dosage) after IMQ application daily, while the MTX group received 1 mg/kg MTX daily, IMQ and NC groups received distilled water. On day 15, the mice were sacrificed by cervical dislocation after narcotized by inhalation of diethyl ether, the blood samples and back skin tissues were collected. The animal experimental procedure was shown in Figure 1.

### Behavior Observation and Psoriasis Area and Severity Index Assessment

During the experiment, body weight of the mice was recorded every day. Behaviors, such as activity, mental state, daily intake of water and food, defecation and urination of the mice were also observed. PASI was used to evaluate the severity of the inflammation in skin. Degrees of erythema, scaling and thickening were scored independently on a scale of 0–4: 0 for none; one for slight; two for moderate; three for severe; four for very severe. The severity of the inflammation was measured by the cumulative score (erythema plus scaling plus thickening) on a scale of 0–12 (van der Fits et al., 2009).

### Histological Analysis

On day 15, the back skin samples were collected and fixed in 10% buffered formalin and embedded in paraffin. Then the samples were cut into 3 µm sections and stained with hematoxylin and eosin (H and E). The samples were examined with an optical microscope (Olympus BX53, Tokyo, Japan). Epidermal thickness was measured in three randomly selected spots.

### Enzyme-Linked Immunosorbent Assay

The serum samples were separated by centrifugation from the blood samples at 12000 g, 4°C for 15 min. Levels of the cytokines IL-17A, IL-23, IL-22, IL-6, TNF-α, IL-1β in serum samples were measured using ELISA kits (Invitrogen, Carlsbad, CA, United States) according to manufacturer’s instructions.

### Quantitative Real-Time PCR Assay

The skin samples were extracted with Trizol reagent (Invitrogen, Carlsbad, CA, United States) to obtain the total RNA. Then the RNAs were reverse transcribed into cDNA with M-MLV reverse transcriptase (Promega, Madison, United States). Quantitative PCR was performed with SYBR Green qPCR Supermix (Invitrogen, Carlsbad, CA, United States) on an ABI 7500 real-time quantitative PCR system (Applied Biosystems, Foster City, United States). The PCR cycle parameters were as follows: denature at 95°C for 5 min, followed by 40 cycles of 95°C for 15 s (denature) and 60°C for 32 s (annealing). Data were analyzed with the 2^−ΔΔCT^ method. Primers used were listed in Table 2.

**TABLE 2:** Primers used for qPCR assays.

Gene	Forward primer	Reverse primer
IL-23	5′-CTC ACC GTG ACG TTT AGG GA-3′	5′-ACT AGA ACT CAG GCT GGG CAT C-3′
IL-17A	5′-TCA TGT GGT GGT CCA GCT TTC-3′	5′-GAA GGC CCT CAG ACT ACC TCA A-3′
IL-22	5′-TTT CCT GAC CAA ACT CAG CA-3′	5′-CTG GAT GTT CTG GTC GTC AC-3′
IL-6	5′-AGA GTA GTG AG GAA CAA GCC-3′	5′-TAC ATT TGC CGA AGA GCC CT-3′
GAPDH	5′-GGT TGT CTC CTG CGA CTT CA-3′	5′-TGG TCC AGG GTT TCT TAC TCC-3′

### Western Blotting

Total protein was extracted from skin samples with RIPA buffer (Beyotime Biotechnology, Shanghai, China). After determination of the protein concentration by BCA kit (Jiancheng Bioengineering Institute, Nanjing, Jiangsu, China), the protein was separated by 10% sodium dodecyl sulfate-polyacrylamide gel electrophoresis (SDS-PAGE) and transferred onto polyvinylidene fluoride membranes (0.45 mm, Millipore, Boston, MA, United States). After blocking with 5% fat-free milk, the membranes were incubated with primary antibodies against NF-κB p65, IκB, p38 MAPK, JNK, ERK, p-p38 MAPK, p-JNK, p-ERK and STAT3 (dilution ratio of 1:1,000, Cell Signaling Technology, Danvers, United States) at 4°C overnight. After washing, the membranes were incubated with horseradish peroxidase (HRP) conjugated secondary antibodies with dilution ratio of 1:4,000 at 37°C for 1 h. Then the membranes were washed for 3 times with Tris buffer saline containing 0.1% (v/v) Tweens 20, and incubated with enhanced chemiluminescence reagent (Millipore, Boston, MA, United States). The signals were captured with X-ray films and quantified with the ImageJ software (NIH, Bethesda, MD, United States). GADPH was used as internal reference protein.

### Statistical Analysis

All data were expressed as mean ± standard deviation (SD). Data were analyzed with SPSS 20.0. Statistical significance was assessed using the one-way analysis of variance (ANOVA). Differences were considered statistically significant at *p* < 0.05.

## Results

### Analysis of the Chemical Constituents of DB Extract

**FIGURE 2:**
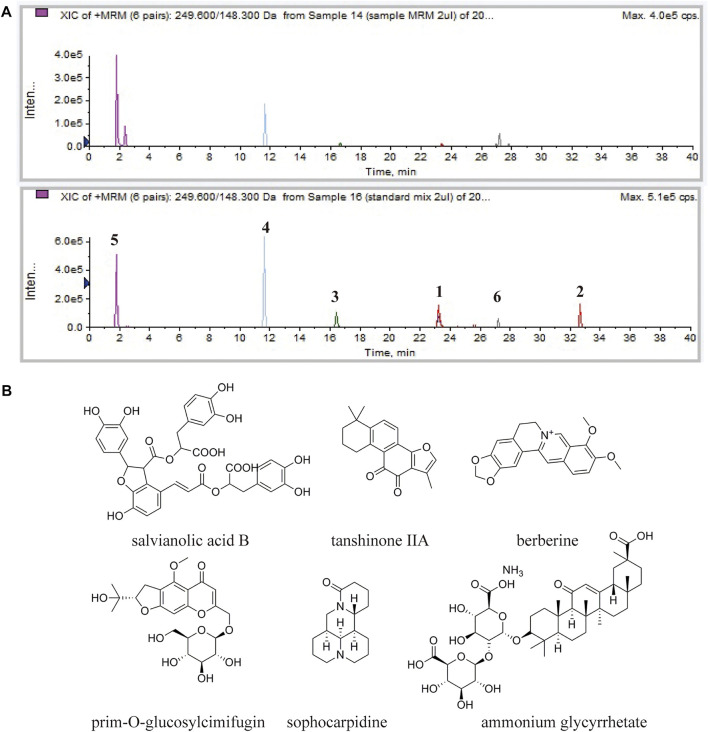
HPLC-MS/MS analysis of the chemical constituents in the DB. **(A)** MRM chromatogram of DB **(upper)** and MRM chromatogram of the referential substances **(lower)**. Salvianolic acid B (1), tanshinone IIA (2), berberine hydrochloride (3), prim-O-glucosylcimifugin (4), sophocarpidine (5) and ammonium glycyrrhetate (6). **(B)** Chemical structures of salvianolic acid B, tanshinone IIA, berberine hydrochloride, prim-O-glucosylcimifugin, sophocarpidine and ammonium glycyrrhetate.

In TCM theory, ingredients in the formula could be divided into four categories, as monarch, minister, assistant and guide drugs. Salvia miltiorrhizae radix et rhizome was the monarch drug of the formula. Phellodendri amurensis cortex and Sophorae flavescentis radix were the minister drug, Saposhnikoviae radix was the assistant drug, and Glycyrrhizae radix et rhizome was the guide drug. These ingredients are the representatives of the four categories in the DB formula, so reference compounds of these ingredients were chosen in the analysis of DB. MRM chromatograms of both DB and referential substances were shown in Figure 2. The retention times of salvianolic acid B, tanshinone IIA, berberine hydrochloride, prim-O-glucosylcimifugin, sophocarpidine, and ammonium glycyrrhetate were 23.2, 32.7, 16.6, 11.6, 1.8 and 27.2 min, respectively. The concentrations of these six components in DB were 12.3, 0.13, 6.71, 3.04, 49.5, 5.96 μg/mg.

### DB Ameliorated IMQ-Induced Psoriasis-like Skin Lesions in Mice

**FIGURE 3:**
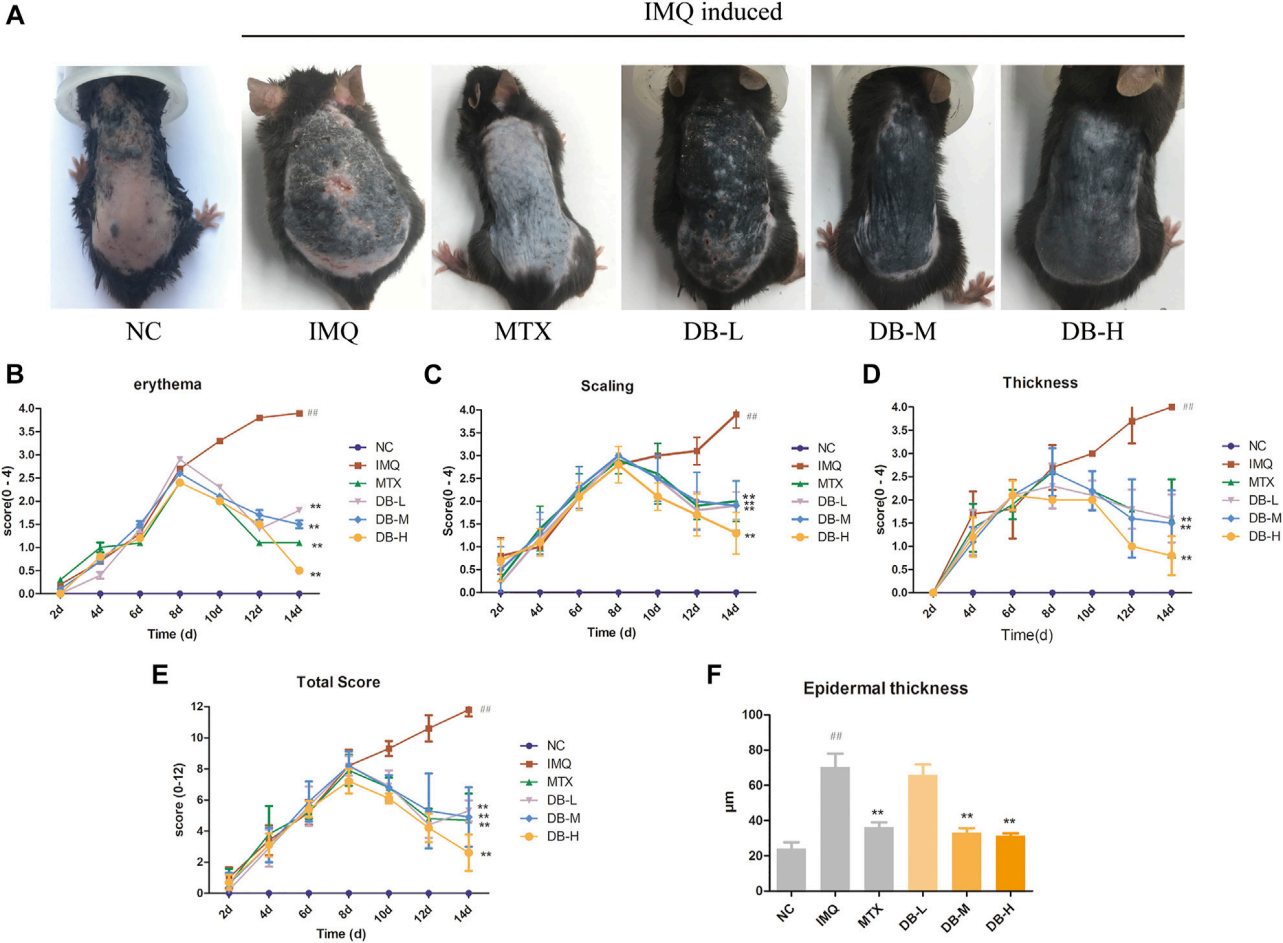
Effects of DB treatment against the psoriasis-like symptoms. **(A)** Images of psoriatic skin lesions in six different groups. **(B–D)** PASI intensity scores (erythema, scaling, epidermal thickness) of skin lesions in six different groups. **(E)** cumulated PASI scores in six different groups. **(F)** Epidermal thickness of back skin was measured in three randomly selected spots. Values are expressed as mean ± SD (*n* = 10). Where ^#^ *p* < 0.05 and ^##^ *p* < 0.01 versus NC group,^*^ *p* < 0.05 and ^**^ *p* < 0.01 versus IMQ group.

During the experiment, no significant differences in body weight, activity, mental state, daily intake of water and food, defecation and urination were observed between the mice in different groups. Typical signs of erythema, scaling and epidermal thickening appeared after 7 days of IMQ application compared with NC group. From day 8 to day 14, DB treatment significantly attenuated the symptoms of psoriasis compared with IMQ-group (Figure 3A).

PASI score was determined for assessing the severity of skin inflammation for all mice. In Figures 3B–E, the PASI scores continually increased after IMQ application from day 1 to day 7. However, the PASI scores decreased gradually after MTX or DB treatment from day 8 to day 14. The PASI scores of DB and MTX-treated groups were significantly lower compared to IMQ group (*p* < 0.01).

### Histopathological Analysis

**FIGURE 4:**
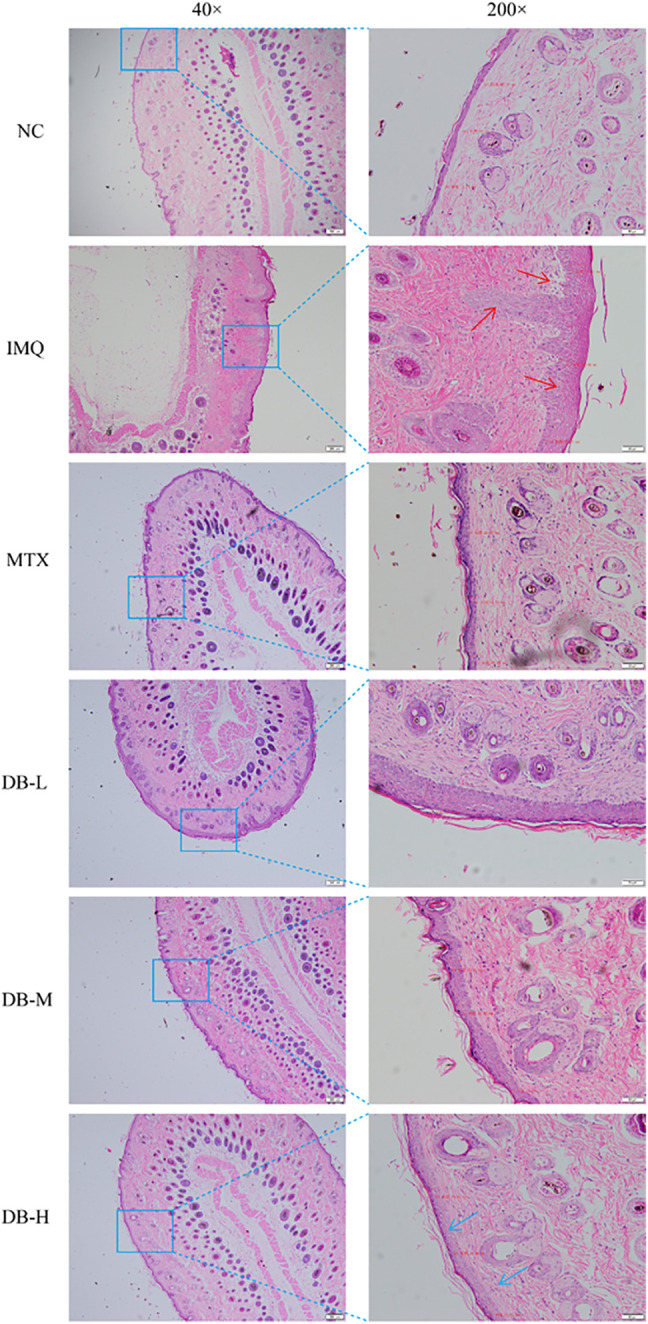
Histopathological images of back skins of the mice (images magnified × 40 and × 200).

Histological changes in the skin tissues after DB treatment were evaluated with H and E staining. In Figure 4, the IMQ-treated group displayed remarkable elevation in epidermal hyperplasia, hyperkeratosis, acanthosis and inflammatory cells’ infiltration. After application of DB, the psoriatic symptoms were alleviated significantly, especially in the high-dosage DB treated group. The epidermal thickness of the mice was measured in three randomly selected spots. In Figure 3F, the IMQ-treated group showed significant thickening of *epidermis* compared to the NC group (*p* < 0.01), while epidermal thickness was significantly reduced in DB and MTX-treated groups (*p* < 0.01).

### DB Decreased the Levels of Inflammatory Cytokines in the Peripheral Blood of IMQ-Induced Psoriatic Mice

**FIGURE 5:**
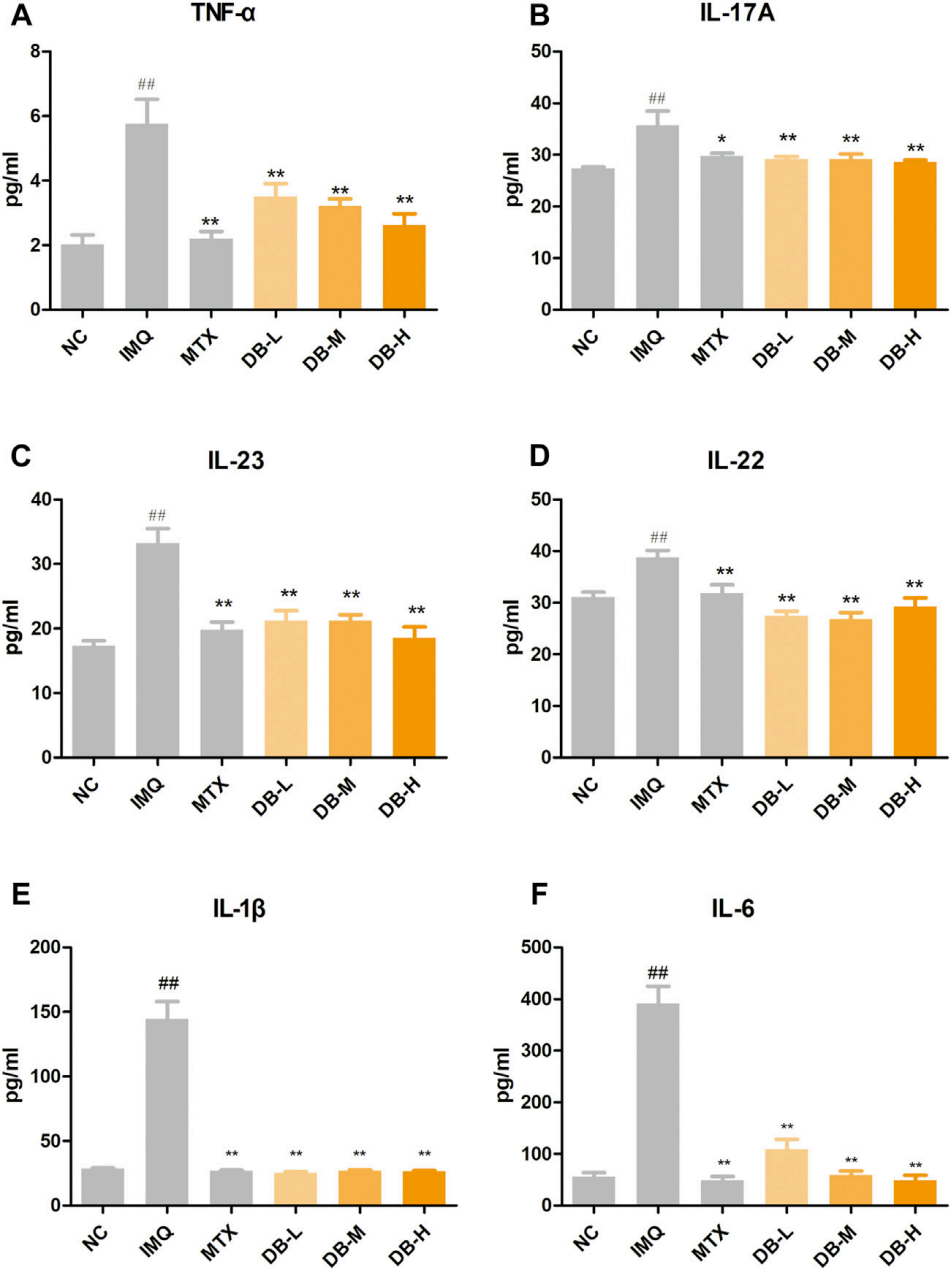
Effects of DB on serum levels of the inflammatory cytokines in IMQ-induced psoriatic mice. The concentrations of TNF-α **(A)**, IL-17A **(B)**, IL-23 **(C)**, IL-22 **(D)**, IL-6 **(E)**, IL-1β **(F)** were measured with ELISA. Values are expressed as mean ± SD (*n* = 10). Where ^#^ *p* < 0.05 and ^##^ *p* < 0.01 versus NC group,^*^ *p* < 0.05 and ^**^ *p* < 0.01 versus IMQ group.

ELISA assays were used to evaluate the effects of DB on the levels of the cytokines in serum. According to Figures 5A–F, TNF-α, IL-17A, IL-23, IL-22, IL-1β, IL-6 levels were significantly increased in the IMQ group compared to NC group (*p* < 0.01). DB treatment significantly decreased levels of these cytokines compared to IMQ group (*p* < 0.01). Levels of TNF-α, IL-23 and IL-6 were decreased with a dose-dependent manner.

### DB Down-Regulated the mRNA Expressions of Inflammatory Cytokines in IMQ-Induced Psoriatic Mice

**FIGURE 6:**
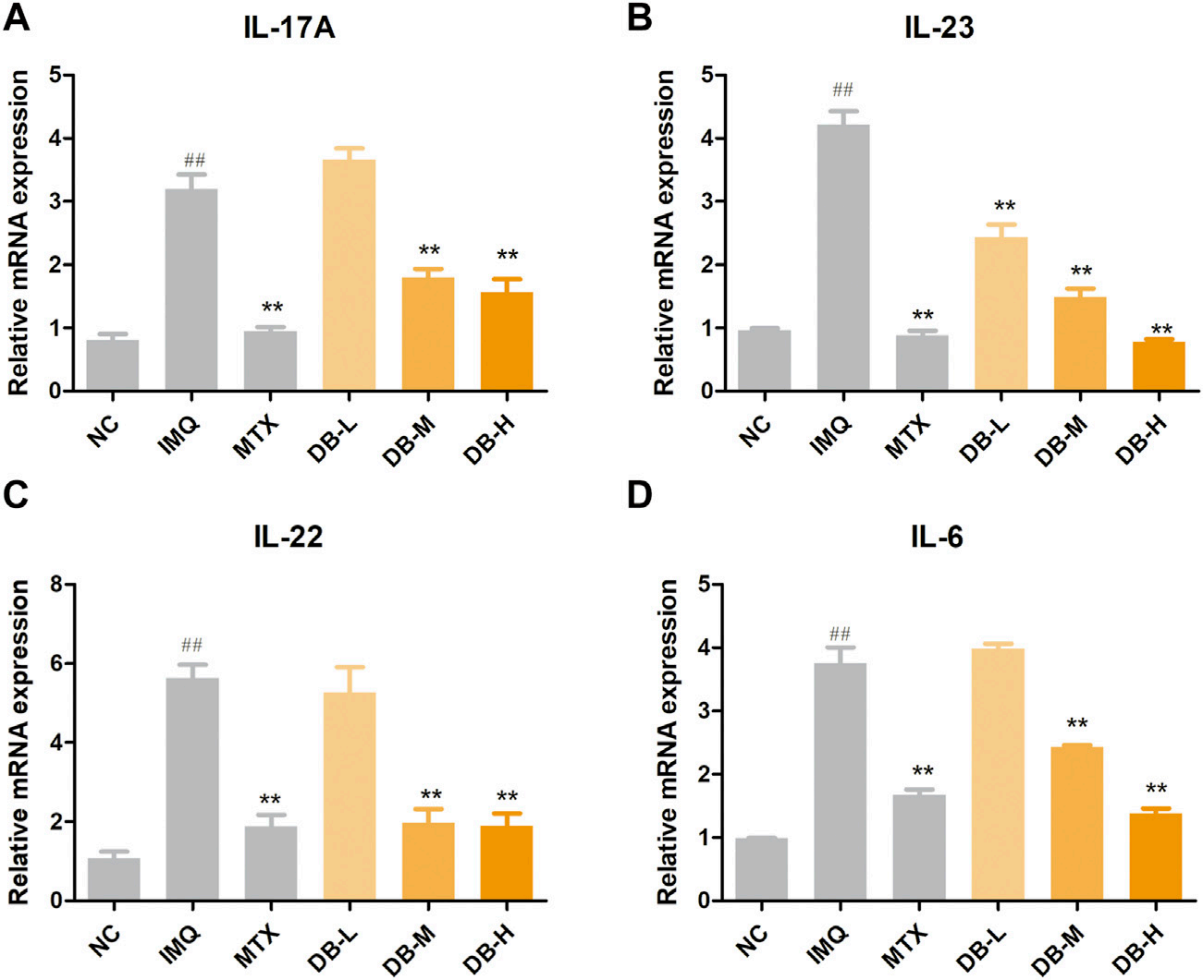
DB reduces the mRNA expressions of IL-17A **(A)**, IL-23 **(B)**, IL-22 **(C)** and IL-6 **(D)** in IMQ-induced psoriatic mice. The mRNA levels in the back skins were detected by real-time PCR. Values are expressed as mean ± SD (*n* = 3). Where ^#^ *p* < 0.05 and ^##^ *p* < 0.01 versus NC group, ^*^ *p* < 0.05 and ^**^ *p* < 0.01 versus IMQ group.

The mRNA expressions of inflammatory cytokines in skin tissues after DB treatment were evaluated by real-time PCR. In Figures 6A–D, IMQ treatment significantly increased the mRNA expressions of IL-17, IL-23, IL-22 and IL-6 compared with NC group (*p* < 0.01). However, the mRNA levels of these cytokines dose-dependently declined in the DB-treated groups (*p* < 0.01).

### DB Inhibited the Activations of NF-κB, MAPKs and STAT3 Signaling Pathways

**FIGURE 7:**
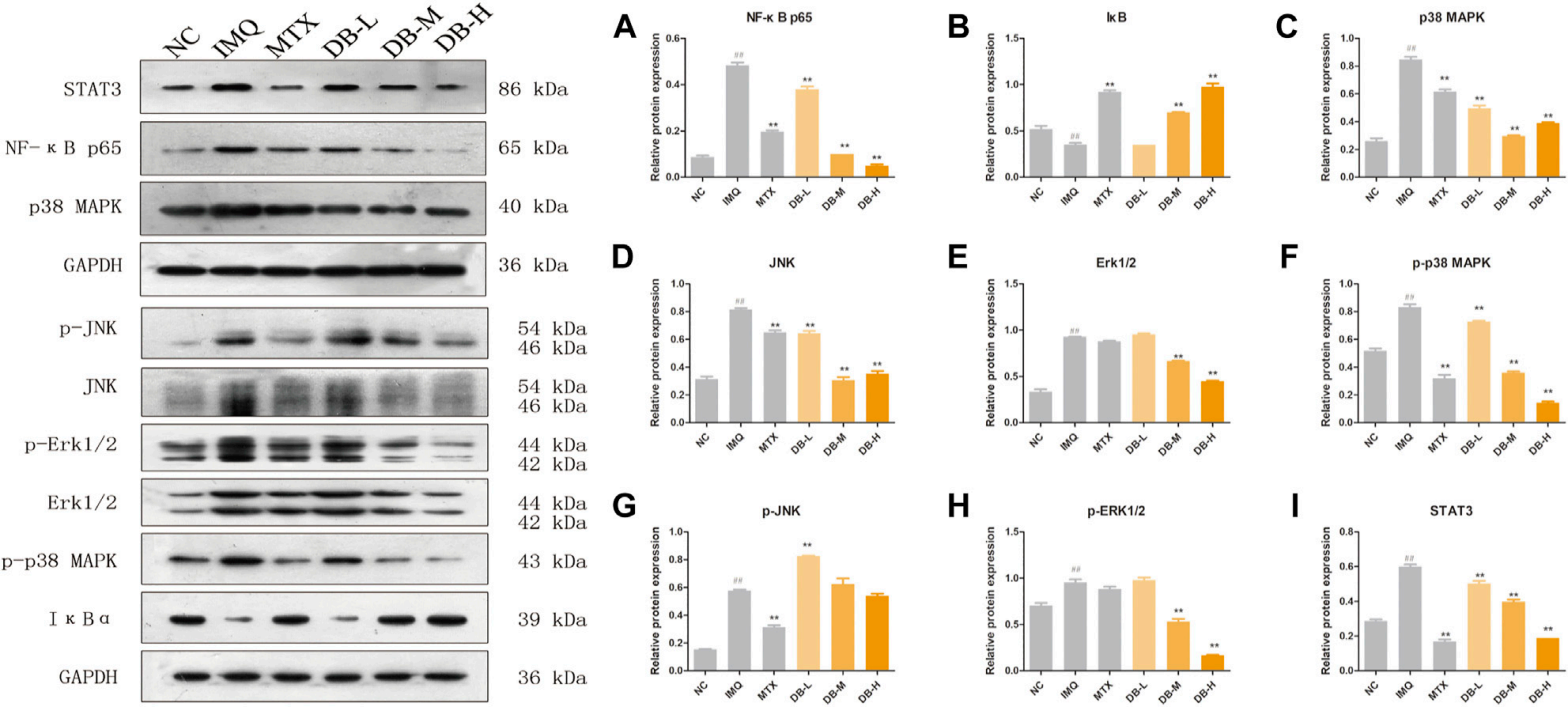
Effects of DB on the NF-κB (NF-κB p65, IκB*α*), MAPKs (p38, JNK, ERK1/2) and STAT3 signaling pathways. The expression levels of p65 **(A)**, IκB*α* **(B)**, p38 **(C)**, JNK **(D)**, ERK1/2 **(E)**, p-p38 **(F)**, p-JNK **(G)**, p-ERK1/2 **(H)**, STAT3 **(I)** in the back skin tissues were detected using western blotting. Values are expressed as mean ± SD (*n* = 3). Where ^#^ *p* < 0.05 and ^##^ *p* < 0.01 versus NC group, ^*^ *p* < 0.05 and ^**^ *p* < 0.01 versus IMQ group.

Western blotting analysis were performed to evaluate the expression levels of the proteins related to NF-κB, MAPKs and STAT3 signaling pathways in the skin tissues after DB treatment. As shown in Figures 7A,B, the expressions of NF-κB p65 were up-regulated and the degradation of IκB was increased after IMQ application compared to the NC group (*p* < 0.01). DB significantly suppressed the expressions of NF-κB p65 and the degradation of IκB in a dose-dependent manner (*p* < 0.01).

In Figures 7C–E, IMQ treatment increased the expressions of p38, JNK and ERK significantly (*p* < 0.01), and these IMQ-induced enhancements in the expressions were markedly prevented by DB (*p* < 0.01). Meanwhile, DB suppressed the phosphorylation of p38 and ERK (*p* < 0.01), though the phosphorylation of JNK were not suppressed by DB (Figures 7F–H).

In Figure 7I, IMQ up-regulated the expression level of STAT3 (*p* < 0.01) while the expression of STAT3 was suppressed significantly after DB treatment in a dose-dependent manner (*p* < 0.01).

## Discussion

Psoriasis is one of the most common inflammatory skin disorders, affecting an estimated 125 million people worldwide (Armstrong and Read, 2020). As a systemic autoimmune disease, psoriasis may also affect nails, joints and other organs, resulting in poor quality of life (Greb et al., 2016). Moreover, people with psoriasis are at higher risk for comorbidities such as obesity, cardiovascular disease, metabolic syndrome and depression. The pathogenesis of psoriasis is complex and not fully understood. Both genetic factors and environmental factors are thought to be involved in the development of psoriasis (Kamiya et al., 2019). Current medications for psoriasis include topical medicines, oral systemic drugs and biologics (Armstrong and Read, 2020). However, treatment of psoriasis remains a challenge due to the low efficacy, side effects or high costs of the medications. New treatment of efficacy and less side effects should be developed.

Traditional Chinese medicine has a long history for the treatment of psoriasis. The earliest record of psoriasis may date back to Sui Dynasty (581–618 AD) in an ancient medical book named Various Pathogenic Designate Theory (諸病源候論) (Cao et al., 2015). In TCM theory, treatment should be based on syndrome differentiation. For psoriasis, syndrome differentiation should base on blood-related symptoms, and treatment should focus on blood. Traditional Chinese doctors differentiate psoriasis into three basic syndromes including blood-heat, blood-dryness and blood-stasis (Deng et al., 2006; Li et al., 2021). Different formulae were formed based on the syndrome differentiation and clinical medication experiences. DB was developed by a famous TCM doctor specialized in skin diseases, and has been efficiently used to treat psoriasis for 30 years in Wuhan Hospital of Traditional Chinese and Western Medicine. DB contains ingredients which have the efficacy to clear blood heat, moisten dryness, promote blood circulation and remove blood stasis, thus DB is used to treat three basic syndrome differentiations of psoriasis (Lin et al., 2005). However, the pharmacological effects and underlying mechanisms of DB remain unclear. In this study, we tried to investigate the anti-psoriatic effects of DB on IMQ-induced psoriasis-like mice model and elucidate the possible underlying mechanisms of DB.

IMQ, an agonist of toll-like receptor (TLR) 7/8, can induce psoriasis on mice skin. IMQ-induced psoriasis-like mice model closely resembles human psoriasis, which critically depends upon the IL-23/TH-17 cytokine axis (van der Fits et al., 2009; Bocheńska et al., 2017). After 7 days of IMQ application, all the mice developed prominent characteristics of psoriasis. After DB treatment, the psoriasis-like symptoms including erythema, white scales, wrinkles and thicken skins, were ameliorated visibly (Figure 3A). The PASI scores of the mice treated with DB decreased significantly compared to the IMQ-group (Figure 3E). Furthermore, histological analysis revealed smoother, fewer inflammatory cells’ infiltration and thinner *epidermis* in DB-treated groups (Figure 4). These results suggested that DB showed obvious anti-psoriatic effects in IMQ-induced psoriatic mice.

The pathogenesis of psoriasis involves the interaction of a variety of cell types, including dendritic cells, macrophages, keratinocytes and T cells (Georgescu et al., 2019; Rendon and Schäkel, 2019). Typically, upon aberrant activation, dendritic cells secrete pro-inflammatory mediators, such as IL-23, IL-6 and TNF-α. These pro-inflammatory mediators induce the proliferation and activation of T cells, such as TH1, TH22 and TH17 cells, which leads further production of cytokines, such as TNF-α, IL-17 and IL-22. These cytokines lead to the recruitment of additional inflammatory cells into the lesion, proliferation and activation of keratinocytes, and other hallmark features of psoriasis. Among these pathways, IL-23/TH17 axis is thought to play a predominant role (Hawkes et al., 2018). Upon stimulation of IL-23, TH17 cells are differentiated from naive T cells and produce IL-17, IL-22, and TNF-α. IL-17 and IL-22 induce proliferation of keratinocytes and release of cytokines from keratinocytes, such as TNF-α, IL-6 and IL-17, which can act back on the DCs and T cells to form a positive feedback loop (Lowes et al., 2014; Deng et al., 2016). TNF-α is a pro-inflammatory cytokine, produced by different cell types including T cells, DCs, macrophages and keratinocytes. TNF-α can induce the production of cytokines to amplify inflammation in psoriasis (Boehncke and Schön, 2015). IL-1β and IL-6 also contribute on the differentiation and activation of Th17 (Parmar et al., 2017). In our present study, application of DB significantly reduced the serum levels of TNF-α, IL-17A, IL-23, IL-22, IL-1β and IL-6 in the DB treated groups compared with the IMQ group (Figure 5). mRNA levels of IL-17A, IL-23, IL-22 and IL-6 were also suppressed by DB compared with the IMQ group (Figure 6), which are in consistent with the ELISA experiments. These results showed that DB could inhibit psoriatic inflammation by regulating the IL-23/TH-17 axis.

Recently, roles of several signaling pathways in pathogenesis of psoriasis have attracted increased attention. NF-κB is a transcription factor, mediating in a variety of inflammatory pathways, cellular proliferation and differentiation, and apoptosis. Under normal conditions, the NF-κB dimer p50/p65 is located in the cytosol and bound to IκB proteins. When NF-κB is activated, IκB is phosphorylated and degraded, p50/p65 is accumulated in the nucleus and activates transcription of target genes. In psoriasis, numerous cell types, including DCs, T cells and keratinocytes, are dependent on NF-κB signaling pathway. NF-κB activates the expressions of TNF-α, IL-6 and IL-1β, and induces inflammatory cells’ infiltration (Pasparakis, 2012; Goldminz et al., 2013; Rai et al., 2020). In our study, DB significantly down-regulated the expressions of NF-κB p65 and up-regulated the expressions of IκB in a dose-dependent manner compared with IMQ group (Figure 7), which suggested that DB could suppress the expressions of NF-κB and degradation of IκB, thus to suppress the activation of NF-κB signaling pathway.

STAT3 is another key transcription factor involved in psoriasis. STAT3 mediates the signal of most cytokines in pathogenesis of psoriasis, including IL-23, IL-17A, IL-22. These cytokines rely on STAT3 pathways to induce the proliferation and activation of TH-17 and keratinocytes. In fact, STAT3 hyperactivation has been found in numerous cell types involved in psoriasis (Calautti et al., 2018; Li et al., 2019a). In our investigation, DB significantly decreased the expressions of STAT3 compared with IMQ group (Figure 7). This result showed DB had ability to suppress STAT3 pathways, especially at high dosage.

Emerging data suggests MAPK signaling pathways play a role in the development of psoriasis. MAPKs, including p38, JNK and ERK signaling pathways, regulate a variety of cellular activities, including cell proliferation, gene expression, apoptosis and inflammation. MAPKs are activated by phosphorylation, and phosphorylate downstream target proteins. MAPKs are reported to be related to the differentiation of Th1 and Th2, secretion of TNF-α, IL-6 and IL-1β, and excessive proliferation of keratinocytes (Mavropoulos et al., 2013; Hammouda et al., 2020). In our present study, except for p-JNK, the expressions of p38, JNK, ERK, p-p38 and p-ERK were significantly decreased by DB (Figure 7). These results indicated that DB could regulate the expressions of p38, JNK, ERK and suppress the phosphorylation of p38 and ERK to inhibit the activations of MAPK signaling pathways.

TCM are thought to have the character of multi-targets and multiple-pathways due to complex constituents. Some ingredients or chemical constituents of DB were reported to have anti-inflammatory activities. For example, salvianolic acid B, major active compounds in Salvia miltiorrhizae radix et rhizome, can suppress NF-κB triggered by TNF-α and the phosphorylation of JNK and ERK1/2 (Dong et al., 2016). Berberine, main active compounds in Phellodendri amurensis cortex, inhibits the inflammatory factors (IL-1, IL-6, COX-2, iNOS) and NF-κB, MAPK signaling pathway to suppress the inflammation (Lin and Lin, 2011; Zou et al., 2017). Cimicifugin of Saposhnikoviae radix is used to treat inflammatory diseases for its activities of inhibition of Th17 and suppression of JAK-STAT and NF-κB signaling pathways (Rahal et al., 2013). Though the whole is not the sum of its parts, the anti-inflammatory activities of ingredients partially explain the anti-psoriatic activity of DB. The therapeutic action of TCM prescription was the whole function of the ingredients. Therefore, we evaluated the anti-psoriatic effects of the whole formula in this study. As the monarch drug of the formula, anti-psoriatic effect of Salvia miltiorrhizae or active compounds of Salvia miltiorrhizae has been reported in several literatures. However, the anti-psoriatic effects of other ingredients and what are the main active compounds of DB still need our further investigation.

## Conclusion

**FIGURE 8:**
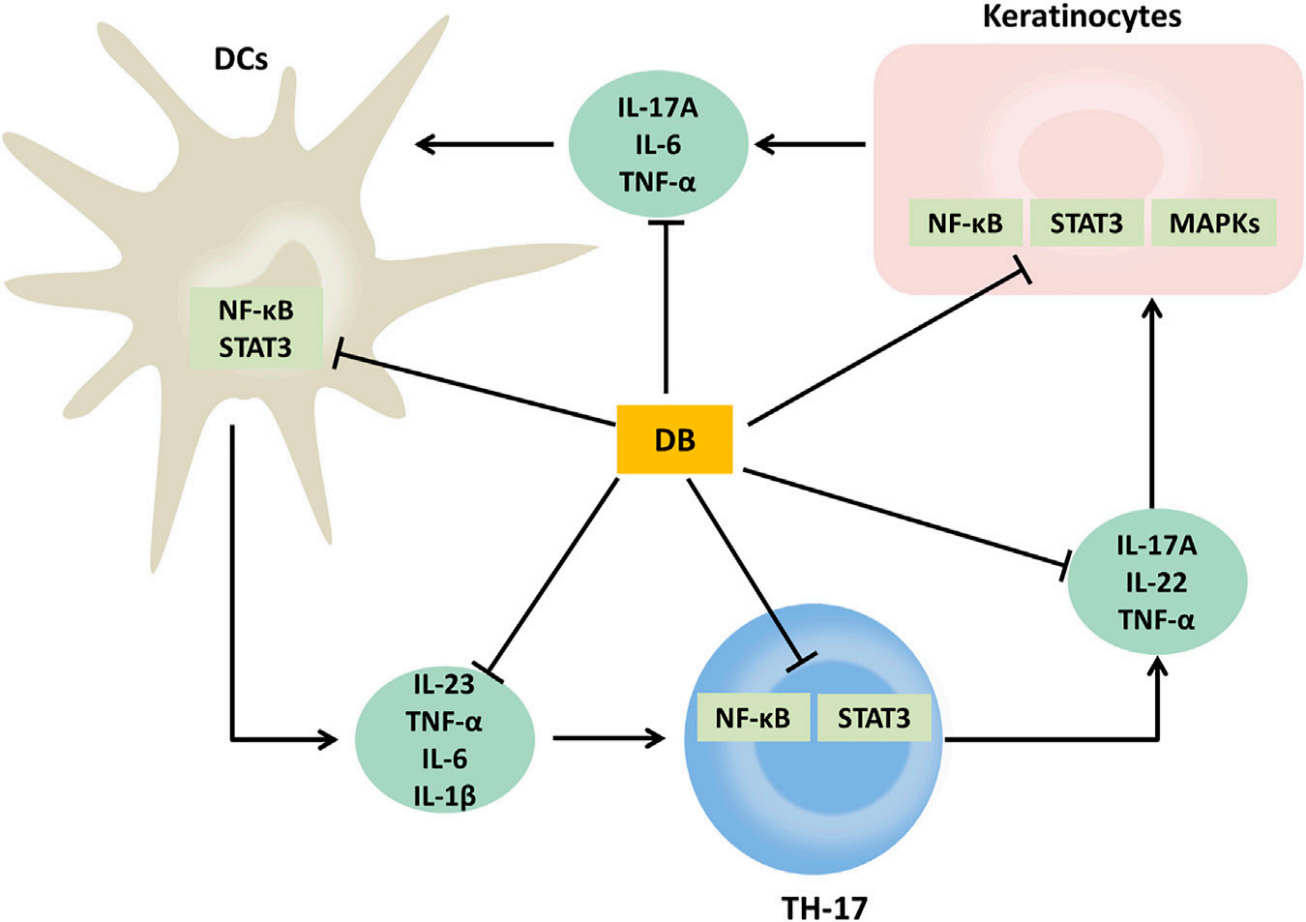
Schematic diagram of proposed mechanisms of the anti-psoriatic effects of DB.

Our results revealed that DB had obvious anti-psoriatic effects in the IMQ-induced psoriatic mice. Its anti-psoriatic effects were related to the regulation of IL-23/TH-17 axis and inhibition on the activation of NF-κB, MAPKs and STAT3 signaling pathways (Figure 8). These findings clarify the therapeutic effects and mechanism of DB, thus provide crucial evidences to support the clinical use of DB.

## Data Availability

The raw data supporting the conclusion of this article will be made available by the authors, without undue reservation.

## Abbreviations

ANOVA, analysis of variance; cDNA, complementary deoxyribonucleic acid; DB, Danshen Baibixiao; DCs, dendritic cells; ELISA, enzyme-linked immunosorbent assay; ERK, extracellular regulating kinase; GAPDH, glyceraldehyde-3-phosphate dehydrogenase; H&E, hematoxylin and eosin; IL, interleukin; IMQ, imiquimod; JAK, Janus-activated kinase; JNK, c-Jun N-terminal kinases; MAPK, mitogen-activated protein kinase; MTX, methotrexate; NC, normal control; NF-κB, nuclear factor-kappa B; PASI, psoriasis area severity index; PCR, polymerase chain reaction; SD, standard deviation; STAT3, signal transducer and activator of transcription 3; TCM, traditional Chinese medicine; TH, T helper; TLR, toll-like receptor; TNF, tumor necrosis factor; UVA, ultraviolet a; UVB, ultraviolet b.
